# Computational Design of Novel Tau-Tubulin Kinase 1 Inhibitors for Neurodegenerative Diseases

**DOI:** 10.3390/ph17070952

**Published:** 2024-07-16

**Authors:** Shahzaib Ahamad, Iqbal Taliy Junaid, Dinesh Gupta

**Affiliations:** 1 Translational Bioinformatics Group, International Centre for Genetic Engineering and Biotechnology, Aruna Asaf Ali Marg, New Delhi 110067, India; 2 Malaria Biology, International Centre for Genetic Engineering and Biotechnology, Aruna Asaf Ali Marg, New Delhi 110067, India; iqbaltjunaid@gmail.com

**Keywords:** Alzheimer’s disease, pharmacophore, molecular dynamic simulation, tau-tubulin kinase 1, ligand design

## Abstract

The tau-tubulin kinase 1 (TTBK1) protein is a casein kinase 1 superfamily member located at chromosome 6p21.1. It is expressed explicitly in the brain, particularly in the cytoplasm of cortical and hippocampal neurons. TTBK1 has been implicated in the phosphorylation and aggregation of tau in Alzheimer’s disease (AD). Considering its significance in AD, TTBK1 has emerged as a promising target for AD treatment. In the present study, we identified novel TTBK1 inhibitors using various computational techniques. We performed a virtual screening-based docking study followed by E-pharmacophore modeling, cavity-based pharmacophore, and ligand design techniques and found **ZINC000095101333**, **LD7**, **LD55**, and **LD75** to be potential novel TTBK1 lead inhibitors. The docking results were complemented by Molecular Mechanics/Generalized Born Surface Area (MMGBSA) calculations. The molecular dynamics (MD) simulation studies at a 500 ns scale were carried out to monitor the behavior of the protein toward the identified ligands. Pharmacological and ADME/T studies were carried out to check the drug-likeness of the compounds. In summary, we identified a new series of compounds that could effectively bind the TTBK1 receptor. The newly designed compounds are promising candidates for developing therapeutics targeting TTBK1 for AD.

## 1. Introduction

**Chart 1:**
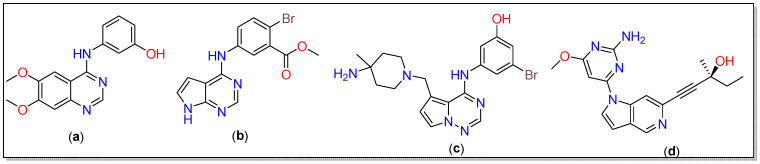
Chemical structure of the inhibitors reported in the literature [13,15,16].

Alzheimer’s disease (AD) is a progressive disorder that causes brain cells to degenerate and die [1,2]. Owing to the damage of neurons, AD causes dementia characterized by declined thinking and disrupted behavioral and social skills [3,4]. Among others, tau-tubulin kinase (TTBK) has been associated with neurodegenerative diseases, including AD, and correlated with cognitive impairment [5]. Among the two isoforms, i.e., TTBK1 and TTBK2, the former is a crucial protein that is over-expressed in AD and is responsible for the phosphorylation and accumulation of the tau protein [6,7]. The tau phosphorylation can be significantly lowered by inhibiting the CNS-specific kinase TTBK1 [8]. TTBK1 belongs to the casein kinase 1 superfamily and is highly expressed in the granular cell layer, the hippocampus, the midbrain cerebellum, and Purkinje cells [9,10]. Tau phosphorylation has been reported at several critical sites within paired helical filaments, including Tyr197, Ser198, Ser199, Ser202, and Ser422. Interestingly, both tau isoforms (TTBK1 and TTBK2) exhibit differences in the phosphorylated amino acid sites. S422 phosphorylation of tau in neurons is attributed to the involvement of TTBK1, leading to neurofibrillary pre-tangles and subsequent tau aggregation [11,12]. Structurally, TTBK1 resembles other kinases in an enriched β strand, an N-terminal domain, and an α-helical C-terminal domain. It has an extended “hinge” region at residues 108–111 that connects both terminals. The P-loop located at residues 40–49 is also a part of the N-terminal domain, while Asp-Phe-Gly (DFG motif) is situated at positions 176–178, and a flexible activation loop (residues 178–202) is located at fragments of the C-terminal [13]. Several TTBK1 inhibitors with activity in the micromolar range have also been reported; yet, no agency has approved them for clinical application owing to poor selectivity and other issues [14,15]. Therefore, the design of new TTBK1 inhibitors is an emerging area of research to tackle neurodegenerative diseases, including AD. In 2013, Xue et al. reported a TTBK1 structure containing quinazoline and pyrrolopyrimidine inhibitors (a and b, Chart 1) [16]. Recently, structurally similar pyrrolopyrimidines were found to reduce TDP-43 phosphorylation, a pathological hallmark of amyotrophic lateral sclerosis (ALS) [10]. By reducing the complexity of the organic core and HBDs and inducing coplanarity in the molecule, Sheriff and co-workers reported the crystal structure of TTBK1 containing a heterocyclic inhibitor 3-({5-[(4-amino-4-methylpiperidin-1-yl)methyl]pyrrolo[2,1-f][1,2,4]triazin-4-yl} amino)-5-bromophenol (c, Chart 1) [13]. Among other interesting structural features, they noted that very little change in the structure of TTK1 occurred upon the binding of the ligand. They concluded that the ligand showed high affinity toward the receptor as it was able to form three good hydrogen bonds, while aromatic cores assisted in hydrophobic interactions. Based on the results obtained, Halkina et al. designed a series of aza indazole-based TTBK1 inhibitors that lower tau phosphorylation in animal models [15]. One of the compounds (d, Chart 1) was found to selectively inhibit TTBK1 with a suitable CNS penetration feature. 

In addition to experimental studies, attempts have been made to design new inhibitors using in silico tools. Recently, Singh et al. [17] carried out a molecular dynamic (MD) simulation and screened the ZINC library to identify the structural profile of the TTBK1–ligand complexes. However, the short time scale (50 ns) was the main drawback of this study as the simulation time is insufficient to provide complete dynamic and conformational details. Considering the gaps in the existing literature and the identification of novel TTBK1 inhibitors, we performed an extensive virtual screening-based molecular docking on 662 million drug-like molecules available in the ZINC database. Pharmacophore screening was conducted to identify the compounds targeting the TTBK1 binding pocket. The ligand designer tool was used to identify the TTBK1 binding pocket and design new ligands (LDs). To understand the behavior of TTBK1 in the presence of identified and designed molecules, we conducted 500 ns MD simulations of the complexes. Several parameters, such as the root mean square deviation (RMSD), root mean square fluctuation (RMSF), solvent accessible surface area (SASA), radius of gyration (Rg), intramolecular hydrogen bond, principal component (PC) analysis, free energy landscape (FEL), and density distribution, have been studied to gain in-depth understanding of this topic. The density functional theory (DFT) calculations also identified the molecular features of the shortlisted and designed compounds, leading to a better understanding of their reactivity and stability.

## 2. Results and Discussion 

The discovery of novel drug candidates is a complex and challenging process that requires a thorough understanding of molecular interactions. Molecular docking-based VS was performed to identify the best-scoring compounds. VS was performed on 662 million drug-like molecules in the ZINC database. After careful analysis, we selected only 3511 molecules out of 15,130 compounds with binding affinity < −8 kcal/mol. These molecules demonstrated low XPG scores and favorable binding interactions, making them the best choices for our needs. In addition to this, the ligand 2KC (3-({5-[(4-amino-4-methylpiperidin-1-yl)methyl]pyrrolo[2,1-f][1,2,4]triazin-4-yl}amino)-5-bromophenol) present in PDB: 4NFN was used as the reference compound. In the upcoming sections, we discuss the results of pharmacophore modeling and ligand design, which enabled us to identify the most effective compounds in the database and design new drug candidates. For clarity, we discuss the results of molecular docking-based VS in each sub-section. 

### 2.1. Pharmacophore Modeling

Pharmacophore modeling is a highly sophisticated tool that is pivotal in drug discovery. It is used to identify the key features that allow a molecule to interact with a biological target. There are two main types of pharmacophore modeling: ligand-based and structure-based [18]. Ligand-based modeling involves the comparison of active ligands, while structure-based modeling uses receptor information to create the pharmacophore hypotheses. The power of pharmacophore modeling lies in its ability to facilitate a wide range of activities, including virtual screening, lead optimization, and the design of new drugs with superior effectiveness and specificity [19,20]. By leveraging the insights provided by pharmacophore modeling, drug discovery researchers can make informed decisions, optimize outcomes, and ultimately develop life-changing treatments that benefit us all. X-ray structure studies on complexes containing heterocyclic inhibitors revealed that the small molecules orient themselves to the ATP pocket and interact via H-bonding with the hinge region (e.g., Gln 108 and Gln110), catalytic lysine (Lys 63), and other regions (Arg119, Phe177, and Glu77) of TTBK1. Our VS-based docking studies and MD simulation results also demonstrated the role of these residues in the stabilization of the complexes (vide infra).

#### 2.1.1. E-Pharmacophore Modeling

**Figure 1:**
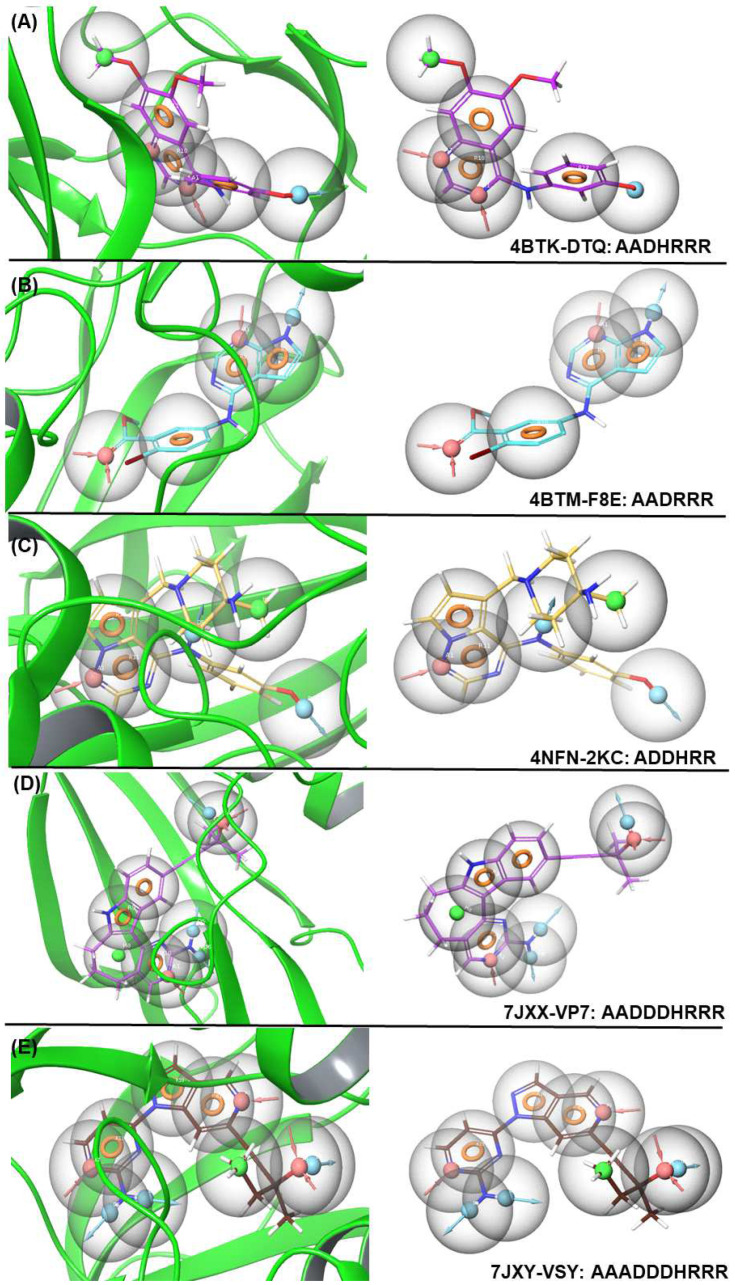
E-pharmacophore models for co-crystal ligands 4BTK-DTQ, 4BTM-F8E, 4NFN-2KC, JXX-VP7, and 7JXY-VSY and their features (**A**–**E**). The graphs were generated using the Schrödinger molecular modeling software version 12.8.117 [21].

E-pharmacophore modeling focuses on the electronic properties of molecules, such as hydrogen bonding, electrostatics, and aromatic interactions. It identifies key electronic features like hydrogen-bond donors/acceptors, positively/negatively charged regions, and aromatic moieties. Moreover, E-pharmacophores are useful for ligand-based drug design, where molecular interactions are crucial for the binding affinity. In this study, we applied various pharmacophore hypotheses to identify drug-like molecules in a library of 3511 molecules (Table S1). This generated a list of hits sorted by fit value, among which the hits with a fitness score > 1.5 were selected for further study. The validation of the E-pharmacophore was calculated and is depicted in Table S1. The distance between each pharmacophore feature of 4BTK-DTQ: AADHRRR, 4BTM-F8E: AADRRR, 4NFN-2KC: ADDHRR, 7JXX-VP7: AADDDHRRR, and 7JXY-VSY: AAADDDHRRR are given in Table S2 and Figure 1. The E-pharmacophore features and hypothesis screening of the shortlisted compounds and the features that provided the best hits are listed in Table 1. The E-pharmacophore modeling identified three potential ligands: one based on a pyrazole core (ZINC000095101333) and the other two based on a diphenylamine core (ZINC000009936617 and ZINC001209984530), which is an established fragment in AD drug design [10].

#### 2.1.2. Cavity-Based Pharmacophore Modeling 

Cavity-based pharmacophores emphasize the geometric and steric features of binding sites or active pockets in proteins. The residues within the binding site of the TTBK1 protein encompass residues 40–48 and 63, while residue 154 is located within the active site. It identifies spatial arrangements of features like hydrophobic regions, hydrogen-bond donors/acceptors, and aromatic rings within the binding site. Furthermore, the cavity-based pharmacophores are essential for structure-based drug design, especially in identifying molecules that fit well in specific protein binding sites. In the TTBK1 protein, two residues (Asp154 and Lys63) are essential for ligand binding (Figure S1A). Features such as distance from A to D (Å), A to A (Å), A to R (Å), D to R (Å), D to D (Å), and R to R (Å) are mentioned in Table S3 and Figure S1B. Modeling using this technique yielded the top three compounds, i.e., ZINC000892508112, ZINC000012184325, and ZINC001243164470 (Tables S4 and S5). Unlike the candidates identified in the previous section, three compounds identified by this technique exhibited a lower docking score but were higher than the reference compound 2KC (−6.83 kcal/mol). Among the candidates identified, ZINC000892508112 showed a score of −8.15 kcal/mol and was stabilized by four H-bonds formed with Ile40, Gln108, Gln110, and Asn113 residues (Figure S2). Compound ZINC000012184325 formed one H-bond with Gln110, leading to a docking score of −8.01 kcal/mol. Finally, compound ZINC001243164470 formed four H-bonds with the residues Glu51, Gln110, Gly111, and Asn113, leading to a docking score of −7.42 kcal/mol.

**Figure 2:**
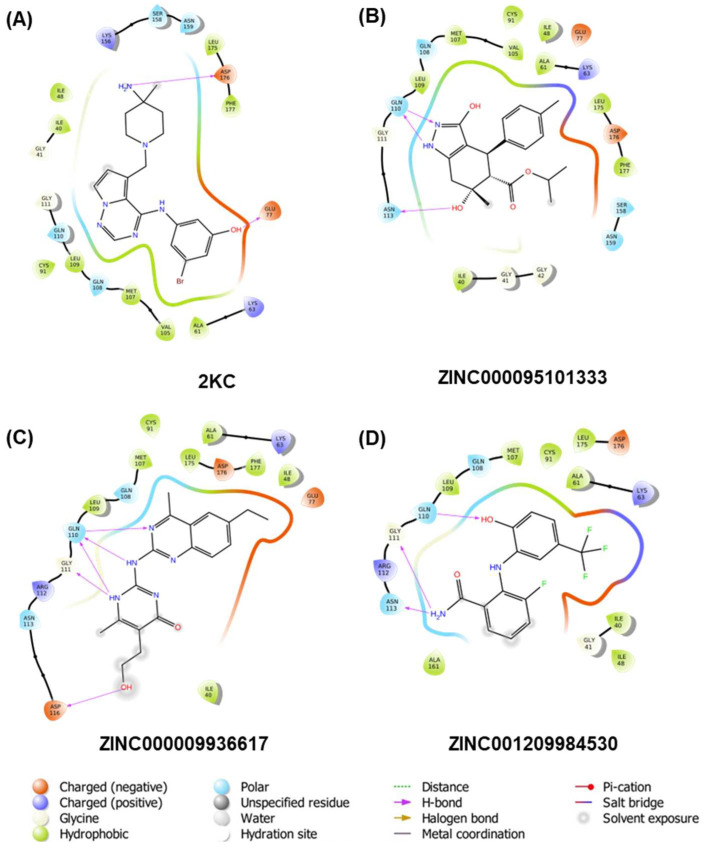
Two-dimensional interaction plots of the co-crystal ligand 2KC and top three compounds shortlisted viz. ZINC000095101333, ZINC000009936617, and ZINC001209984530 (**A**–**D**, respectively).

Molecular docking studies were conducted on the receptor TTBK1, and the results indicated that three compounds exhibited a higher affinity than the reference compound 2KC (−6.83 kcal/mol). Specifically, compound ZINC000095101333 showed a docking score of −8.64 kcal/mol, while ZINC000009936617 and ZINC001209984530 showed scores of −8.94 and −8.27 kcal/mol, respectively. Figure 2 depicts the 2D plots of the interactions between the receptors and the three compounds, while the interacting residues are given in Table 2. As shown in the figure, the reference compound formed five H-bonds, the compounds ZINC000095101333 and ZINC001209984530 formed three H-bonds. Contrarily, compound ZINC000009936617 formed five H-bonds with the receptor. 

Furthermore, the Molecular Mechanics/Generalized Born Surface Area (MMGBSA) values were computed for the co-crystal ligand 2KC and the compounds ZINC000095101333, ZINC000009936617, ZINC001209984530, ZINC000892508112, ZINC000012184325, and ZINC001243164470. The calculated values were −36.85, −48.26, −44.19, −44.14, −48.37, −49.15, and −41.71 kcal/mol, respectively (as shown in Table 2). These calculations offer valuable insights into the binding affinities of the selected molecules, shedding light on their potential effectiveness. This information is of utmost importance in evaluating the potential of these compounds as effective binders in the field of drug discovery.

### 2.2. Ligand Design via Scaffold Hopping

Scaffold hopping is a crucial strategy in drug design, aimed at modifying the core scaffold of the 2KC molecule while preserving its overall pharmacological properties. This process involves replacing a lead compound’s central structural motif or scaffold with a different scaffold while maintaining or improving its desired biological activity and other drug-like properties. The rationale behind scaffold hopping lies in overcoming limitations associated with the original scaffold, such as poor bioavailability, toxicity, or lack of patentability. We explore alternative chemical space by switching to a new scaffold and potentially discovering compounds with improved potency, selectivity, or other desirable properties.

**Figure 3:**
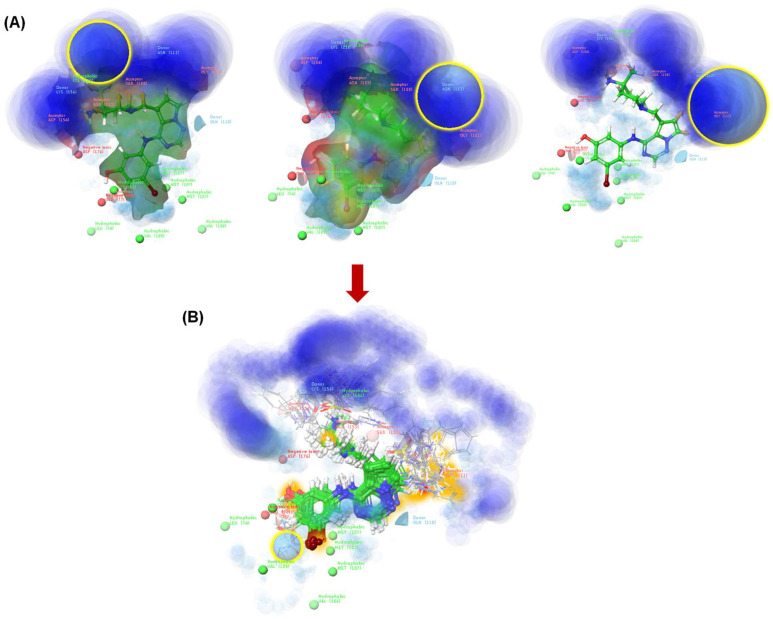
The TTBK1 active site (blue circular region). The ligand design determines the location of H-bond donors and acceptors (**A**), and after the generation of the newly designed molecules, the blue color is less apparent (**B**). The plots were generated using the Ligand Designer module of Schrödinger software [21].

We identified a lead 2KC compound with the desired biological activity (Figure 3). This compound served as the starting point for scaffold hopping. The lead compound’s scaffold was analyzed to identify key pharmacophore elements and regions contributing to its activity. This analysis helped select an appropriate replacement scaffold that retains essential interactions with the target protein. Using ligand design tools within Schrödinger software, the original scaffold was systematically replaced with alternative scaffolds from a database of chemical fragments or scaffolds. The software evaluated the compatibility of each replacement scaffold with the target binding site and predicted the resulting binding affinity and other relevant properties. The designed compounds were subjected to virtual screening against the target protein using molecular docking methods. This step helped to identify promising candidates with favorable binding modes and predicted activity. 

**Figure 4:**
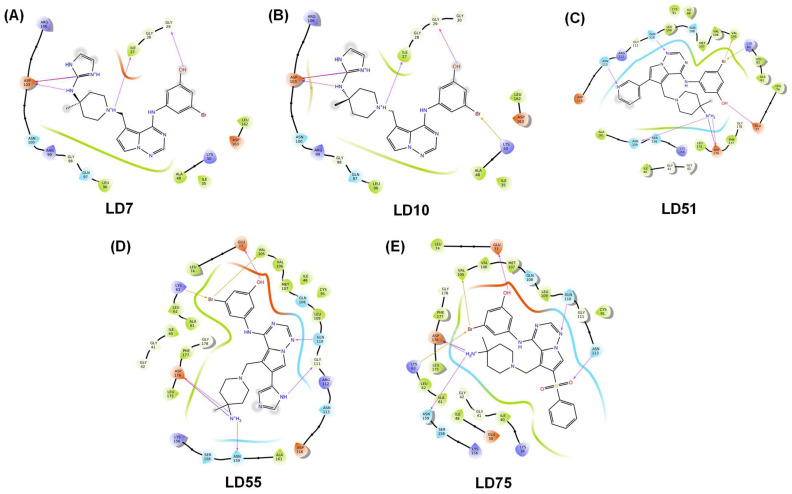
Docking (2D) plots of the top five newly designed compounds LD7, LD10, LD51, LD55, and LD75 (**A**–**E**, respectively). The H-bonds are represented in the pink arrow.

The active site of the TTBK1 protein is shown in blue (Figure 3A). The merged 2KC protein-ligand complex is selected, and a growth space grid is generated, depicted by the yellow circle. This illustrates the area for modifying or adding substitutions to design new ligands. Additionally, the light blue regions indicate parts that can be modified for protein interactions (Figure 3B). The studies suggest that the active sites represent the H-bond donor and acceptor sites. It reveals that the molecules bearing donor and acceptor atoms bind in a particular zone, as highlighted in blue, which would be active. Based on the results of pharmacophore modeling, we designed 115 new-generation molecules via scaffold hopping. Among the designed compounds, LD7, LD10, LD51, LD55, and LD75 were more potent and, therefore, selected for further studies. The details of the designed compounds, such as class, docking scores, and interacting residues, are given in Table 3 and Figure 4. The docking score of the compounds can be ranked in the following order: LD7 (−10.37 kcal/mol) > LD75 (−10.10 kcal/mol) > LD10 (−9.98 kcal/mol) > LD51 (−9.87 kcal/mol) > LD55 (−9.79 kcal/mol). The highest docking score of the compound LD7 may be attributed to multiple polar interactions involving an -NH_2_^+^ unit (present between the imidazole ring and piperidine ring) and Asp176/Asn159 fragments of the receptor. The fused pyrimidine nitrogen formed a polar bond with Gln110. The hydroxyl group of the benzene ring formed polar bonds with Glu77. However, the bromine atom (Br) of the benzene ring formed a polar bond with Lys63 and Val105. 

In the case of the second most potent compound, i.e., LD75, we observed that an ammonium (-NH_3_^+^) cation is attached at position 4 of piperidine without an imidazole ring and formed an H-bond with Asp176 and Asn159. Benzene sulfoxide was introduced at the five-member ring of the fused pyrimidine ring in which the oxygen atom of sulfoxide formed a polar bond with Asn113. This may be why it displays a higher score than LD10 and LD55, but a benzene ring at the five-member ring, which is a hydrophobic group, may diminish the docking score more than LD7. The rest of the polar bonds are the same in both molecules. LD10, bearing an amino-bridged imidazolium ring, interacted with Asn159 and Asp176 via polar interactions. The imidazole/imidazolium group is absent in the LD51 compound; only the -NH_3_^+^ cation is present and forms polar bonds. It was observed that compound LD55 consists of an imidazole group at five members of the fused pyrimidine ring and showed the least docking score. Based on these observations, we conclude that the imidazole group attached to the charged nitrogen atoms is necessary, followed by the sulfoxide group at the five-member ring of the fused pyrimidine ring to be active. The MMGBSA values were determined for the LD7, LD10, LD51, LD55, and LD75 molecules, yielding MMGBSA values of −46.71, −39.43, −54.02, −57.00, and −50.75 kcal/mol, respectively (Table 3).

### 2.3. Pharmacological and ADME/T Properties

The ADME/T and pharmacological properties are crucial for selecting and prioritizing drug candidates. The output of the QikProp module indicated that the compounds showed no violation in the properties, whereas LD51 and LD75 exhibited only one violation (high molecular weight), which is acceptable (Tables S6 and S7). The blood–brain barrier penetration ranges from −0.575 to −1.991, logP ranges from 4.31 to 5.17, QPlogKhsa ranges from 0.26 to 1.25, SASA ranges from 709 to 732, and log Kp ranges from −2.09 to −3.60 (Table S7). Further evaluation using the Lipinski RO5 rule revealed that the compounds showed a molecular weight range of 400–620 Da, hydrogen-bond acceptor (HBA) range of 4–11, and hydrogen-bond donor (HBD) range of 0 to 2 (Table S7). The results revealed that the compounds were within the acceptable range, indicating that they possess high bioavailability compared to 2KC. However, it is also worth mentioning that the recommended value for QPlogBB falls between −3.0 and 1.2, but in our case, the values were between −0.268 and −1.521, which might be due to the varying polar fragments. Similarly, the QPlogHERG value for the compounds was below −5, indicating a possible toxic nature. These data should be considered while designing drugs based on the shortlisted compounds. 

### 2.4. MD Simulation

**Figure 5:**
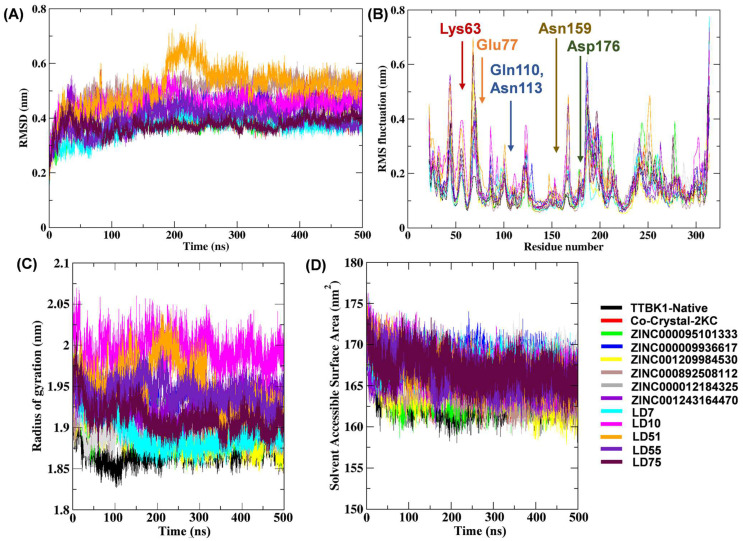
Plots depicting the RMSD (**A**), RMSF (**B**), Rg (**C**), and SASA (**D**) of the native TTBK1 protein and complexes. The results of the MD simulation were visualized using the GROMACS 5.18.3 software package [22].

We performed MD simulations to understand the complex stability and interaction profiles of the most promising compounds inside the active site of TTBK1. The MD simulation results at 500 ns of native TTBK1, TTBK1-reference molecules (2KC), TTBK1-ZINC000095101333, TTBK1-ZINC000009936617, TTBK1-ZINC001209984530, TTBK1-ZINC000892508112, TTBK1-ZINC000012184325, TTBK1-ZINC001243164470, TTBK1-LD7, TTBK1-LD10, TTBK1-LD51, TTBK1-LD55, and TTBK1-LD75 are depicted in Figure 5. The structural parameters, including RMSD, RMSF, Rg, SASA, and FEL, were evaluated as a function of time and are discussed below.

#### RMS Deviation and RMS Fluctuations 

The docked complexes were subjected to RMSD analysis to assess the residual flexibility of TTBK1. The average RMSD values of the native molecules (TTBK1), complexes with reference molecules (2KC), ZINC compounds, and newly designed compounds (LD) were in the range of 0.36–0.51 nm. All complexes showed steady RMSD (~0.4 nm) values, except LD51, which showed a slightly higher value (RMSD ~0.55 nm: Table 4 and Figure 5A). On the other hand, compounds such as ZINC000095101333, LD7, and LD75 exhibited lower RMSD values, indicating a stable bond with TTBK1. This is supported by the RMS deviation in Cα-atoms, which remained stable, with significant differences in the values of all three complexes. 

To determine the flexible and rigid regions within the complexes, we analyzed their RMSF values. The average RMSF values of the complexes with ZINC compounds were in the range of 0.13–0.17 nm, while it was 0.14–0.18 nm for compounds of the LD series. These values were comparable to the reference compound 2KC, which showed an average RMSF value of 0.15 nm, which was higher than that of the native protein (0.14 nm). Overall, complexes with lower average RMS fluctuations than the native protein (RMS of the native protein = 0.14 nm) were noted in the LD75 (0.14 nm), LD7 (0.14 nm), and ZINC001209984530 (0.13 nm) complexes. These findings suggest that these compounds exhibit fewer atomic fluctuations and higher stability (Figure 5B). Furthermore, the impact of molecular docking on the dynamic behavior of TTBK1 protein residues was shown by comparing the RMSF profiles of the native protein to those of its complexes with top-performing ligands, including ZINC000095101333, LD7, LD55, and LD75. This analysis provides insights into how ligand binding influences the flexibility and stability of key residues within the protein. The orthosteric binding sites of the TTBK1 showed that the co-crystal ligand 2KC interacted with the residue Lys63 and formed hydrogen bonds with the binding site residue as well as Glu77, Gln110, Asn159, and Asp176 amino acids. It is noteworthy that the native TTBK1 exhibited a fluctuation of approximately 0.071 nm at Lys63, while the TTBK1–2KC complex showed a fluctuation of 0.070 nm in the RMSF plot. Furthermore, for the compound ZINC000095101333, the RMS fluctuations at Gln110 and Asn113 were analyzed. Gln110 showed an RMSF value of 0.083 nm in the native protein, which increased to 0.107 nm in the complex, indicating a significant fluctuation peak. Asn113 exhibited RMSF values of 0.075 nm in the native protein and 0.074 nm in the complex, indicating minimal fluctuation. Compound LD7 interacted with Glu77, Gln110, Asn159, and Asp176 residues in the docked complex. In the RMSF plot, we observed a fluctuation (0.091 nm) in Glu77, which reached 0.095 nm in the native protein. The fluctuation in Gln110 was 0.083 nm in the native protein, which increased to 0.091 nm in the LD7 complex. Asn159 showed a fluctuation of 0.079 nm in the native protein and 0.078 nm in the LD7 complex. Asp176 exhibited a fluctuation of 0.093 nm in the native protein and 0.078 nm in the LD7 complex. On the other hand, the LD55-TTBK1 docked complex showed interactions within the binding site pocket involving residues such as Glu77, Gln110, Asn113, Asn159, and Asp176. The RMSF plot showed notable changes in residue fluctuations upon ligand binding. Specifically, the fluctuation peak of Glu77 increased from 0.095 nm in the native protein to 0.201 nm in the LD55 complex. Gln110 fluctuation also increased from 0.083 nm (native) to 0.110 nm (LD55 complex). An increased fluctuation was also observed in Asn113, from 0.075 nm (native) to 0.091 nm in the LD55 complex. Similarly, an increase in fluctuation can be noted for Asn159 (0.072 to 0.101 nm) and Asp176 (0.093 to 0.102 nm) upon the binding of LD55. In the case of the LD75–TTBK1 complex, the key residues were Glu77, Gln110, Asn113, Asn159, and Asp176. In this particular case, we noted a decreased fluctuation in Glu77 (0.095 nm for the native to 0.092 nm for the complex), Gln110 (0.083 nm for the native to 0.083 nm for the complex), and Asp176 (0.093 nm for the native to 0.091 nm for the complex). However, a slight rise in the fluctuation of residue Asn113 (0.075 nm for the native to 0.077 nm for the complex) and Asn159 (0.072 nm for the native to 0.076 nm for the complex) is also noteworthy.

**Figure 6:**
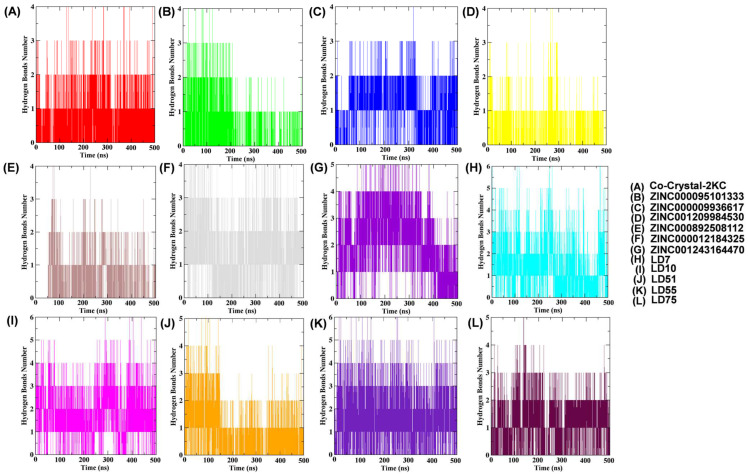
Plots of hydrogen bonds between TTBK1 protein residues and selected ligands over a 500 ns MD simulation were analyzed using GROMACS, providing detailed data for each frame.

Rg examination dictates the compactness of the docked complexes. The results showed that the Rg values of the complexes containing the native, reference, ZINC000095101333, ZINC000009936617, ZINC001209984530, ZINC000892508112, ZINC000012184325, ZINC001243164470, LD7, LD10, LD51, LD55, and LD75 compounds were 1.87, 1.91, 1.90, 1.92, 1.89, 1.90, 1.91, 1.93, 1.89, 1.99, 1.95, 1.94, 1.91, and 1.87, respectively (Figure 5C). It is noteworthy that LD7 and LD75 bound firmly to the active site and maintained the stability and compactness of the protein structure. SASA analysis (Figure 5D and Table 4) revealed stable hydrophobic contacts between the receptor and ligand complexes, like the reference molecules, which make the maximum region of TTBK1 accessible to the solvent molecules. Hydrogen bonds between TTBK1 and the ligands were analyzed using gmx hbond. Figure 6 shows these interactions over the 500 ns simulation. The average number of hydrogen bonds for each complex was calculated as follows: co-crystal-2KC (0.61), ZINC000095101333 (0.42), ZINC000009936617 (1.02), ZINC001209984530 (0.30), ZINC000892508112 (0.49), ZINC000012184325 (1.35), ZINC001243164470 (2.1), LD7 (1.34), LD10 (1.74), LD51 (0.73), LD55 (1.61), and LD75 (1.02). The data indicate that the screened compounds, such as ZINC000009936617, ZINC000012184325, and ZINC001243164470, and newly designed compounds, such as LD7, LD10, LD55, and LD75, were able to interact with the target receptor more intricately than the co-crystallized ligand.

### 2.5. Free Energy Landscape (FEL) Analysis

**Figure 7:**
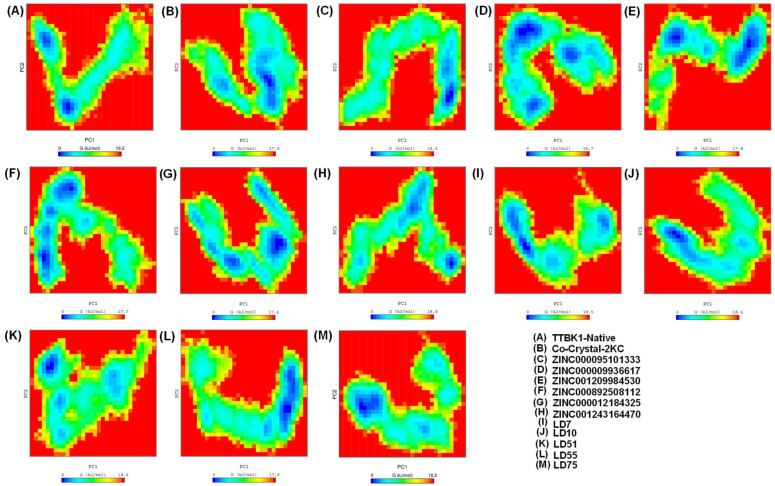
The Gibbs energy landscape (FEL) plot was estimated during a 500 ns MD simulation. The graphs were generated with the help of GROMACS software [22].

The 2D plot depicting a well-defined global energy minima basin provides valuable insights into the conformational changes of the system under study. The contour of the FEL reveals the presence of multiple relative minima, which signifies the structure’s stability and dynamic nature. Remarkably, all the complexes, except for the native protein shown in Figure 7, converged to the lowest energy conformation and basin. Within the binding pocket of the structure, we observed significant conformational changes and motion of the atoms comprising the residues. These findings result from the strong binding of ligands within the active site pocket of the TTBK1 receptor, which causes minimal fluctuations in stability during the trajectory analysis. As a result, our MD simulations consistently show that the co-crystal-2KC, ZINC000095101333, LD7, LD55, and LD75 complexes maintain stable conformations throughout the simulation period. This indicates that they are promising candidates for the inhibition of the TTBK1 receptor and require further research.

### 2.6. Density Functional Theory (DFT) Studies

**Figure 8:**
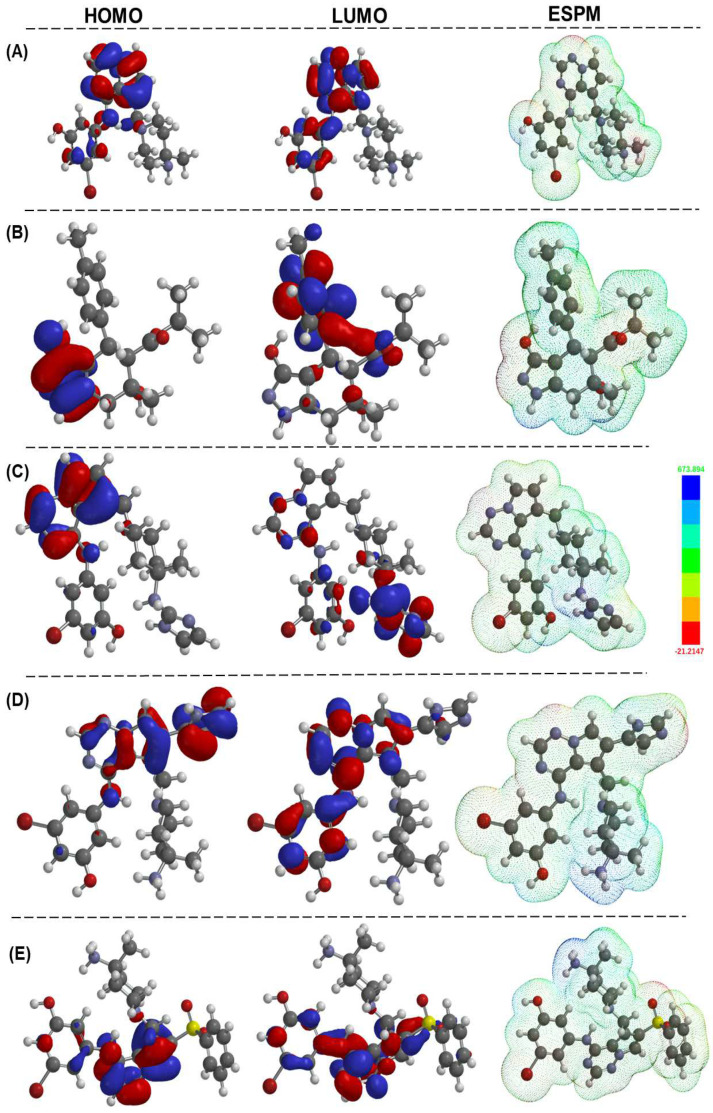
HOMO, LUMO orbitals, and the electrostatic potential map of shortlisted compounds, namely, 2KC, ZINC000095101333, LD7, LD55, and LD75 (**A**–**E**, respectively). The calculations performed in the gas phase were executed using the Spartan20 package with the B3LYP/6-31G* method, and the graphs were generated with the assistance of Spartan20 (Version 20.1.2) software [27].

DFT is a vital tool for drug discovery. With its ability to accurately predict the electronic structure and energy of molecules [23], DFT plays a critical role in understanding molecular interactions with biological targets, optimizing molecular structures, and predicting pharmacological properties. DFT has revolutionized the drug discovery process by facilitating the design of novel therapeutics. Its unparalleled precision and accuracy have made it an invaluable asset for researchers in the field [24]. DFT calculations supplement the structural and photophysical features of small to large organic, inorganic, and organic–inorganic hybrid compounds. Using this tool, one can obtain several pieces of information at the sub-atomic level, which can be correlated with their properties and applications [25,26]. For instance, the frontier molecular orbital (FMO) energy is correlated to the compound’s bioactivity. It can also be utilized to determine its reactivity, kinetic/thermodynamic stability, etc. In the present study, we performed DFT calculations at the B3LYP/6-31G* level in the gas phase using the Spartan 20 program. The study’s findings are given in Figure 8 and Figure S3. DFT studies have found some interesting results for certain classes of molecules. For instance, compounds from the ZINC series exhibit closer HOMO (−5.36 to −6.26 eV) and LUMO (−0.24 to −1.90 eV) va lues, leading to a band gap (ΔE) in the range of 3.76–5.24 eV (Table 5).

On the other hand, all newly designed compounds (LD series) showed a deeper HOMO (−7.67 to −8.16 eV) and LUMO (−3.33 to −3.91 eV). This led to a slight decrease in the overall ΔE value (3.33–3.91 eV), typical for small organic compounds bearing conjugated units. These values also suggest that the compounds possess fair stability (the larger the energy gap, the more significant the stability and, thus, the lower the chemical reactivity will be). As shown in Figure 8, both the HOMO and LUMO are localized over the pyrrolo[2,1-f][1,2,4]triazine core with little contribution from imidazole/phenylsulfonyl and substituted N-phenyl rings.

We also estimated the values of chemical descriptors viz the ionization energy (I.E), electron affinity (E.A), electronegativity (χ), chemical potential (μ), chemical hardness (η), softness (σ), and electrophilicity index (ω) of the compounds. These values, which were calculated using FMO energy, provide clues about a compound’s chemical reactivity, bioactivity, and stability. For example, a negative μ value shows that the compound is stable and cannot decompose spontaneously. In this case, the value of the chemical potential (μ) was relatively higher (−2.86 to −3.80 eV) for the ZINC series than the LD series (−5.50 to −6.00 eV), indicating a higher stability of the latter under normal conditions. Similarly, the electrophilicity index (ω) is related to the stabilization in energy when the system gains an additional electronic charge from the surroundings and quantifies the global electrophilic power of the molecule. Based on the energies of frontier orbitals, the following values were noted for the descriptors: η = 1.88–2.62 eV, σ = 0.19–0.26 eV^−1^, and ω = 1.56–8.91 eV. An important feature is that the values are closer to the ligand-bound co-crystal-2KC, indicating a stark similarity between the designed compounds and co-crystal ligands. Therefore, we strongly believe the designed compounds can serve as potent inhibitors.

## 3. Materials and Methods

### 3.1. Hardware and Software 

The Tyrone workstation (124 GB RAM) was installed with an NVIDIA graphics card (GPU P2000 6 GB) and was utilized for modeling, docking, and DFT calculations. The MD simulation was carried out using the GROningen MAchine for Chemical Simulations (GROMACS) (Version 5.13.3) and was installed on a high-performance computing (HPC) cluster of International Business Machine (IBM) Power9 CPU nodes (total 160 CPUs) with NVIDIA TESLA (model: V100 32 GB) GPUs and a Red Hat Enterprise Linux operating system [28]. 

#### 3.1.1. Protein and Ligand Preparation

**Figure 9:**
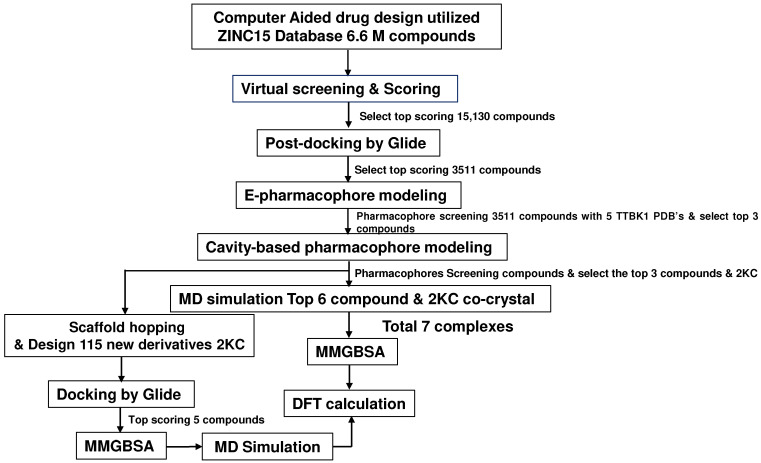
The workflow of this study utilized ZINC15’s 6.6 million active compounds and involved virtual screening. The top-scoring molecules were further utilized for pharmacophore analysis, MD simulations, and DFT calculations.

We used the ZINC15 database (docking.org) [29], a widely used resource in drug discovery, accessed in January 2021. The molecular docking was performed using PDB ID 4NFN, which was chosen due to its favorable binding energies among the five PDB crystal structures of the receptor (PDB IDs: 4NFN, 4BTK, 7JXY, 7JXX, and 4BTM). All PDB files were downloaded from the PDB database [13,15,16]. The TTBK1 structures were prepared using the Maestro’s protein preparation wizard tool. During the preparation, the structures were analyzed for missing loops and atoms [21]. Virtual screening (VS) was performed on 662 million drug-like molecules in the ZINC database using the lead finder module of the Cresset package [29,30]. VS yielded 15,130 top-scoring molecules with a high binding affinity. Furthermore, ligands were prepared and saved in .sdf format to perform post-VS studies using Glide (Maestro) (Figure 9).

#### 3.1.2. Post-VS Receptor Grid Generation

This study utilized 662 million compounds for virtual screening using the PDB structure (PDB ID: 4NFN) with the help of Lead Finder software. For the post-docking process, the compounds were docked using the Glide three-tier docking program, comprising high-throughput virtual screening (HTVS), standard precision (SP), and extra precision (XP) protocols, utilizing the OPLS-5 force field [31,32]. The protein structure generated a receptor grid with cubic dimensions of 15 Å × 15 Å × 15 Å along the X, Y, and Z axes. This grid was positioned at the center of the bound ligand in the TTBK1 binding pocket, aligned with the location of co-crystallized ligands in TTBK1. The default parameters that were used are as follows: scaling factor of 0.8 for van der Waals radii of the nonpolar ligand atoms and partial charge cut-off of 0.15. The protein was fixed, and the ligands were kept flexible during the docking calculations. Finally, the docking protocol was validated by re-docking ligands into the binding pocket. Additionally, the reliability of the docking protocol was assessed by analyzing docking scores and binding affinities to ensure accurate and consistent results. The best-docked conformations of each ligand were selected based on their extra precision Glide (XP) scores and interaction profiles.

#### 3.1.3. E-Pharmacophore Modeling

We selected five PDB structures for the E-pharmacophore modeling study, namely 4NFN, 4BTK, 7JXY, 7JXX, and 4BTM. Protein structures were prepared using the protein preparation wizard of Schrödinger Maestro with an OPLS_2005 force field. The receptor grid generation tool in Maestro, Schrödinger was applied to the design grids of all protein crystal structures concentrated at co-crystal ligands. The co-crystallized ligands were refined utilizing LigPrep in Maestro, Schrödinger with Epik and an OPLS_2005 force field. The pharmacophore sites of the co-crystal ligands were arranged based on their Glide XP docking binding energies and selected for pharmacophore development. The H-bonds, electrostatic bonds, π-π stackings, π-cations, and other interactions were contained within the Glide XP descriptors.

Schrödinger PHASE was applied to generate pharmacophore features like the H-bond donor (D), H-bond acceptor (A), negative ionizable (N), positive ionizable (P), hydrophobic group (H), and aromatic residue (R) based on XP energy descriptors. The H-bond donor (D) and H-bond acceptor (A) of crystal ligands were pictured as vectors directed to the corresponding H-bond acceptors and H-bond donors at the binding site of the protein structures, respectively. The most favorable sites were selected for E-pharmacophore hypothesis development by utilizing the excluded volume. Initially, a maximum of ten pharmacophores were set in Schrödinger PHASE to develop pharmacophore models, which were validated, gradually reduced, and selected as the most suitable model for the pharmacophore-based VS [33]. The Enrichment Factor (EF) was computed to assess the model’s ability to identify active compounds within a vast dataset. A higher EF suggests improved performance. Additionally, the Goodness-of-Hit score was determined to assess the overall effectiveness of the pharmacophore model, considering both sensitivity and specificity.

### 3.2. Receptor Cavity-Based Pharmacophore Modeling and Lead Optimization

The receptor cavity-based VS is a flexible and robust method to screen potent inhibitors for apo receptors. This method involves the consideration of the chemical features of the binding pocket residues of a target protein. First, the target critical pocket residues were determined using the SiteMap module of the Maestro package [34]. The PHASE module employed a multiple-copy simultaneous search method to find energetically favorable positions and properties of molecules to generate a receptor cavity-based pharmacophore model. Subsequently, the resultant residues from SiteMap analysis were used as input to generate the model [35]. Finally, the selected pharmacophore hypothesis was used to search drug-like compounds comprising 3511 molecules from the ZINC database [29]. This screening protocol produced a list of hit molecules ranked by fit value. The hits with fitness scores greater than 1.5 were chosen for further analysis. The best pharmacophore model was validated using various methods, including the external validation of the scoring system. The primary aim of validating a quantitative pharmacophore model is to evaluate its capability to identify active compounds and predict their corresponding molecules. The effectiveness of the models was further evaluated through external validation. The predictive power of the generated model was additionally assessed against a set of 1000 decoy compounds obtained from the DUD-E database. The EF and Robust Initial Enhancement (RIE) were computed to evaluate the model’s reliability and provide an accurate ranking of compounds.

Lead optimization was carried out using the ligand designer technique. In this technique, the merged protein-ligand complexes are selected, and a growth space grid is generated, where light blue regions indicate modifiable parts for protein interactions. Using the workflows menu to enhance ligand-protein interactions, select specific regions for modification, identify target atoms, and form hydrogen bonds. 

On the other hand, the selection of pharmacophore hypotheses and hit molecules was based on key factors to find compounds with potential binding affinity to the target protein. We used Schrödinger’s software to generate pharmacophore hypotheses, focusing on cavity-based ones. These helped us understand how molecules interact with the target protein’s binding site. Hit molecules were chosen based on how well they matched the essential features of the pharmacophore within the binding site cavity. We calculated a fitness score to measure this alignment. A higher score means better alignment and a higher chance of favorable interactions with the protein. We prioritized molecules with higher fitness scores for further investigation as potential TTBK1 inhibitors. These hits underwent more screening and validation steps to confirm their effectiveness. Identifying cavity-based pharmacophore patterns helped us understand how ligands bind to the target protein. They revealed specific features like hydrogen-bond donors and acceptors and hydrophobic regions that are crucial for binding. This insight helped us in designing better drug candidates.

### 3.3. Bioactivity and Toxicity Prediction (Drug-Likeliness and ADMET Study) 

The QikProp in Maestro 12.8.117, Schrödinger 2021-2 was used to predict the hits’ ADME properties and toxicity [36]. QikProp predicts various significant, physicochemical, and pharmaceutically useful descriptors essential for rational drug design [32]. 

### 3.4. MMGBSA Free Energy Calculations

Furthermore, a Prime MMGBSA study was carried out. MM-GBSA free energy calculations were performed using the Schrödinger Prime module. The calculations utilized the GBSA continuum model, with the Gaussian surface representing the SASA. This approach helped us to identify compounds with favorable interactions and binding poses based on their binding free energies (ΔG). The use of the Gaussian surface for the SASA ensures a more accurate representation of solvation effects, enhancing the reliability of the binding affinity predictions. 

### 3.5. MD Simulations 

MD simulations were conducted with the GROMACS 5.18.3 software package [22]. The topology generation for TTBK1 utilized the GROMOS9643a1 force field [37]. Molecular topologies for the drug molecule were created using the PRODRG server due to the unavailability of the GROMACS package [28,38,39]. The systems were solvated in a cubic box using the simple point charge model (SPC/E). To maintain a 0.15 M concentration, counter ions (Na^+^ and Cl^−^) were added for system neutralization. Energy minimization was performed on all neutralized systems using a combination of steepest descent and conjugate gradients (50,000 steps each).

System equilibration was achieved through the NVT (regulation of volume) and NPT (regulation of pressure) ensembles. The NVT ensemble maintained a constant temperature of 300 K, while the NPT ensemble maintained a constant pressure of 1 bar. The SHAKE algorithm was employed to constrain the equilibrium distances of hydrogen atoms, and periodic boundary conditions were applied. Long-range electrostatic forces were defined using the Particle Mesh Ewald (PME) method with a cut-off of 1.0 nm for van der Waals and columbic interactions [40]. The bonds and angles were constrained using the LINC algorithm. Production runs were conducted for each complex with a duration of 500 ns and a time integration step of 10 ps. Energy, velocity, and trajectory updates were performed at specified intervals. The dynamic behavior of protein–ligand complexes was assessed by calculating the Cα-atom deviations in the protein.

### 3.6. DFT Calculations

Spartan 20 software is primarily a molecular mechanics software package commonly used for DFT calculations. Herein, we utilized DFT calculations for molecules derived from ZINC and ligand design techniques with a B3LYP/6-31G* level of theory. The simulation of molecular behavior in the gas phase allowed the study of the fundamental properties of molecules, such as their geometry, electronic structure, and energetics [27,41].

## 4. Conclusions

This study employed various techniques, including virtual screening, post-VS analysis, E-pharmacophore modeling, cavity-based pharmacophore modeling, and ligand design techniques to screen and identify novel TTBK1 inhibitors. Eleven compounds (six screened and five newly designed compounds) were shortlisted, leading to the identification of **ZINC000095101333**, **LD7**, **LD55**, and **LD75** as potential TTBK1 inhibitors. MD simulation studies on a 500 ns scale were carried out, and RMSD, RMSF, and FEL analyses were performed. Finally, pharmacological and ADME/T studies were also carried out to evaluate the potential of the identified candidates. Based on the results obtained, we speculate that our designed compounds could serve as the scaffolds for drug discovery against AD and other neurodegenerative diseases.

## Figures and Tables

**Table 1:** Pharmacophore features and hypothesis screening of 3511 shortlisted compounds.

PDB ID	Hypothesis	Pharmacophore Features	No. of Hits	ZINC ID
4BTK	AADHRRR	A: Aromatic Ring (two features) D: Donor Atom (hydrogen-bond donor) H: Hydrophobic Region R: Rotatable Bond (three features)	369	ZINC000095101333, ZINC001209984530, and ZINC000095101335
4BTM	AADRRR	A: Aromatic Ring (two features) D: Donor Atom (hydrogen-bond donor) R: Rotatable Bond (three features)	1169	ZINC000009936617, ZINC000017180924, and ZINC000095101333
4NFN	ADDHRR	A: Aromatic Ring D: Donor Atom (two features) H: Hydrophobic Region R: Rotatable Bond (two features)	819	ZINC000095101333, ZINC000020798038, and ZINC001209984530
7JXX	AADDDHRRR	A: Aromatic Ring (two features) D: Donor Atom (three features) H: Hydrophobic Region R: Rotatable Bond (three features)	643	ZINC000009936617, ZINC000017180924, and ZINC001209984530
7JXY	AAADDDHRRR	A: Aromatic Ring (three features) D: Donor Atom (three features) H: Hydrophobic Region R: Rotatable Bond (three features)	83	ZINC000095534860, ZINC000428178776, and ZINC000095101333

**Table 2:** Docking score and interacting residues of the compounds identified by modeling techniques.

ZINC ID	3D Structure	Docking Score (kcal/mol)	MMGBSA (kcal/mol)	Interacting Residues	
				H-Bonded	Others
Co-crystal ligand 2KC	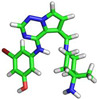	−6.83	−36.85	Lys63, Glu77, Gln110, Asn159, and Asp176	Lys38, Ile40, Gly41, Gly42, Ile48, Glu50, Ala61, Leu62, Cys91, Val105, Val106, Met107, Gln108, Leu109, Gly111, Asn113, Ser158, Ala161, Leu175, and Phe177
ZINC000095101333	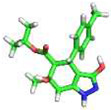	−8.64	−48.26	Gln110 and Asn113	Ile40, Gly41, Gly42, Ile48, Ala61, Lys63, Glu77, Cys91, Val105, Met107, Gln108, Leu109, Gly111, Ser158, Asn159, Leu175, Asp176, and Phe177
ZINC000009936617	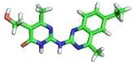	−8.94	−44.19	Gly111, Arg112, and Asp116	Ile40, Ile48, Ala61, Lys63, Glu77, Cys91, Met107, Gln108, Leu109, Arg112, Asn113, Leu175, Asp176, and Phe177
ZINC001209984530	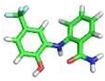	−8.27	−44.14	Gln110, Gly111, and Asn113	Ile40, Gly41, Ile48, Ala61, Lys63, Cys91, Met107, Gln108, Leu109, Arg112, Ala161, Leu175, and Asp176
ZINC000892508112	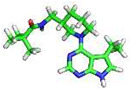	−8.15	−48.37	Ile40, Gln108, Gln110, and Asn113	Lys38, Gly41, Gly42, Ile48, Glu50, Ala61, Lys63, Glu77, Cys91, Met107, Leu109, Gly111, Arg112, Ser158, Asn159, Ala161, Leu175, Asp176, and Phe177
ZINC000012184325	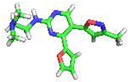	−8.01	−49.15	Gln110	Lys38, Ile40, Gly41, Gly42, Ile48, Glu50, Ala61, Lys63, Glu77, Val105, Met107, Gln108, Leu109, Gly111, Arg112, Asn113, Ser158, Asn159, Ala161, Arg164, Leu175, Asp176, Phe177, and Gly178
ZINC001243164470	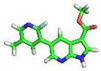	−7.42 kcal/mol	−41.71	Glu51, Gln110, Gly111, and Asn113	Ile40, Gly41, Ile48, Ala61, Lys63, Glu77, Cys91, Met107, Leu109, Gly111, Asn113, Ser158, Asn159, Ala161, Leu175, Phe177, and Gly178

**Table 3:** Docking score, MMGBSA, and interacting residues of the newly designed compounds.

Code	3D Structure	Docking Score (kcal/mol)	MMGBSA (kcal/mol)	Interacting Residues	
				H-Bonded	Others
LD7	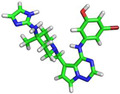	−10.37	−46.71	Glu77, Gln110, Asn159, and Asp176	Ile40, Gly41, Gly42, Ile48, Ala61, Leu62, Lys63, Leu74, Cys91, Val105, Val106, Met107, Gln108, Leu109, Gly111, Asn113, Asp154, Lys156, Ser158, Ala161, Leu175, Phe177, and Gly178
LD10	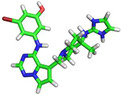	−10.10	−39.43	Glu77, Gln110, Asn159, and Asp176	Ile40, Gly41, Gly42, Ile48, Ala61, Leu62, Lys63, Leu74, Cys91, Val105, Val106, Met107, Gln108, Leu109, Gly111, Arg112, Asn113, Lys156, Ser158, Ala161, Leu175, Phe177, and Gly178
LD51	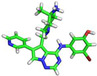	−9.98	−54.02	Glu77, Gln110, Asn113, Asn159, and Asp176	Ile40, Gly41, Gly42, Ile48, Ala61, Leu62, Lys63, Leu74, Cys91, Val105, Val106, Met107, Gln108, Leu109, Gly111, Arg112, Asn113, Asp116, Lys156, Ser158, Ala161, Leu175, Phe177, and Gly178
LD55	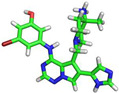	−9.87	−57.00	Glu77, Gln110, Gly111, Asn159, and Asp176	Ile40, Gly41, Gly42, Ile48, Ala61, Leu62, Lys63, Leu74, Cys91, Val105, Val106, Met107, Gln108, Leu109, Arg112, Asn113, Asp116, Lys156, Ser158, Ala161, Leu175, Phe177, and Gly178
LD75	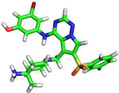	−9.79	−50.75	Glu77, Gln110, Asn113, Asn159, and Asp176	Lys38, Ile40, Gly41, Gly42, Ile48, Glu50, Ala61, Leu62, Lys63, Leu74, Cys91, Val105, Val106, Met107, Gln108, Leu109, Gly111, Lys156, Ser158, Leu175, Phe177, and Gly178

**Table 4:** MD simulation trajectory analysis of the native TTBK1 and the shortlisted compounds.

Compound ID	MD Simulation				
	RMSD (nm)	RMSF (nm)	Rg (nm)	SASA (nm^2^)	H-Bonds
Native TTBK1	0.42	0.14	1.87	163.94	-
Co-Crystal-2KC	0.41	0.15	1.91	166.08	0.61
ZINC000095101333	0.38	0.16	1.90	164.75	0.42
ZINC000009936617	0.42	0.16	1.92	167.53	1.02
ZINC001209984530	0.41	0.13	1.89	164.61	0.30
ZINC000892508112	0.50	0.15	1.90	164.93	0.49
ZINC000012184325	0.41	0.16	1.91	167.34	1.35
ZINC001243164470	0.43	0.17	1.93	166.66	2.10
LD7	0.36	0.14	1.89	167.51	1.34
LD10	0.45	0.18	1.99	167.23	1.74
LD51	0.51	0.18	1.95	167.77	0.73
LD55	0.39	0.15	1.94	166.71	1.61
LD75	0.36	0.14	1.91	166.92	1.02

**Table 5:** Chemical reactivity parameters of the studied compounds from DFT.

Compound S. No.	E_HOMO_ (eV)	E_LUMO_ (eV)	Δ*E* ^a^ (eV)	*I*^b^ (eV)	*A*^c^ (eV)	*χ* ^d^ (eV)	*η*^e^ (eV)	*σ*^f^ (eV^−1^)	*μ*^g^ (eV)	*ω*^h^ (eV)
Co-crystal-2KC	−8.16	−3.85	4.31	8.16	3.85	6.00	2.15	0.23	−6.00	8.36
ZINC000095101333	−5.48	−0.24	5.24	5.48	0.24	2.86	2.62	0.19	−2.86	1.56
ZINC000009936617	−5.87	−1.79	4.08	5.87	1.79	3.83	2.04	0.24	−3.83	3.60
ZINC001209984530	−5.66	−1.90	3.76	5.66	1.90	3.78	1.88	0.26	−3.78	3.80
ZINC000892508112	−5.36	−0.71	4.65	5.36	0.71	3.03	2.32	0.21	−3.03	1.97
ZINC000012184325	−6.08	−1.10	4.98	6.08	1.10	3.59	2.49	0.20	−3.59	2.58
ZINC001243164470	−6.27	−1.34	4.93	6.27	1.34	3.80	2.46	0.20	−3.80	2.89
LD7	−7.98	−3.72	4.26	7.98	3.72	5.85	2.13	0.23	−5.85	8.22
LD10	−7.68	−3.33	4.35	7.68	3.33	5.50	2.17	0.23	−5.50	6.97
LD51	−7.93	−3.88	4.05	7.93	3.88	5.90	2.02	0.24	−5.90	8.60
LD55	−7.67	−3.91	3.76	7.67	3.91	5.79	1.88	0.26	−5.79	8.91
LD75	−8.16	−3.85	4.31	8.16	3.85	6.00	2.15	0.23	−6.00	8.35

a = |E_HOMO_ − E_LUMO_|; b = −E_HOMO_; c = −E_LUMO_; d = (I + A)/2; e = (I − A)/2; f = 1/2η; g = −(I + A)/2; h = μ^2^/2η.

## Data Availability

All generated or analyzed data during this study are included in this article. The raw data from this study can be provided upon reasonable request to the corresponding author.

## Supplementary Materials

The following supporting information can be downloaded at: https://www.mdpi.com/article/10.3390/ph17070952/s1, Figure S1: The binding pocket of TTBK1 protein along active site residues Asp154 and Lys63 (A). The same binding pocket selection for the Cavity-based pharmacophore (B) and top shortlisted screened ligand from the ZINC database (i–iii). These figures were created with the assistance of Schrödinger software. Figure S2: Docked Cavity-based top shortlisted screened ligand (A–C). These figures were created with the assistance of Schrödinger software. Figure S3: HOMO, LUMO orbitals and electrostatic potential map of shortlisted compounds ZINC000009936617, ZINC001209984530, ZINC000892508112, ZINC000892508112, ZINC00012184325, ZINC001243164470, LD10, and LD51 (A–G, respectively). The Calculations were performed by the Spartan’20 package with B3LYP/6-31G* in the gas phase. The graphs were produced with assistance from Spartan20 software (V1.0.0) package. Table S1: The selected hypothesis and the validation of E-pharmacophore model. Table S2: The measurement of distances between e-pharmacophore hypothesis features in Å. Table S3: Distance between the features of cavity pharmacophore hypothesis. Table S4: The top three compounds were retrieved after the pharmacophore model validation and docking. Table S5: Validation of cavity pharmacophore hypothesis. Table S6: Lipinski RO5 of drug likeliness and ADME/T properties of the shortlisted compounds from ZINC database and Co-Crystal ligand interpreting pharmacological parameter. Table S7: Lipinski RO5 of drug likeliness and ADME/T properties of the ligands were compared to reference molecules, interpreting pharmacological parameters and predicted using the QikProp module of Schrodinger.
