# Supplementary material for: Computational Design of Novel Tau-Tubulin Kinase 1 Inhibitors for Neurodegenerative Diseases
Source: Pharmaceuticals (Basel). 2024 Jul 16;17(7):952. doi: 10.3390/ph17070952 (PMC11280166; doi:10.3390/ph17070952)
Supplement: Supplementary file 1 [file pharmaceuticals-17-00952-s001.zip › pharmaceuticals-3043013-supplementary.pdf]

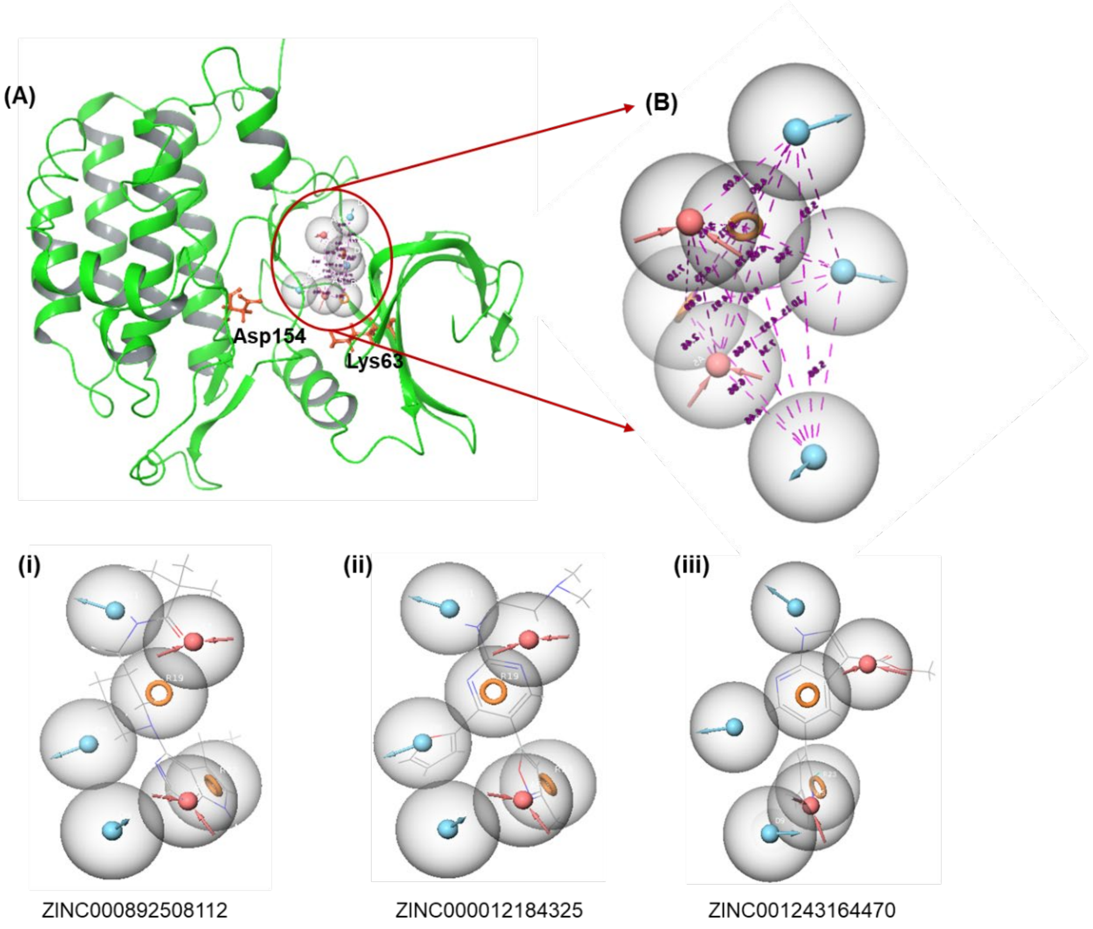

Figure S1. The binding pocket of TTBK1 protein along active site residues Asp154 and Lys63 (A). The same binding pocket selection for the Cavity-based pharmacophore (B) and top shortlisted screened ligand from the ZINC database (**i-iii**). These figures were created with the assistance of Schrödinger software [1].

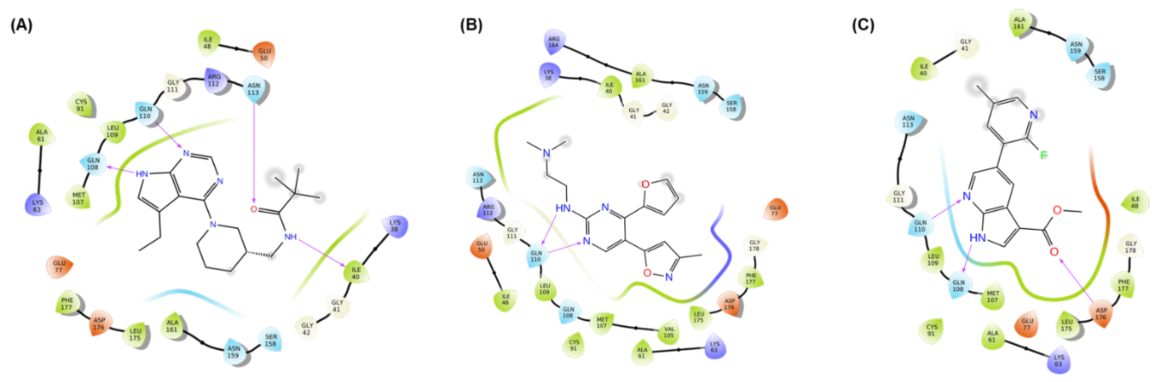

ZINC000892508112 ZINC000012184325 ZINC001243164470

Figure S2. Docked Cavity-based top shortlisted screened ligand (**A-C**). These figures were created with the assistance of Schrödinger software.

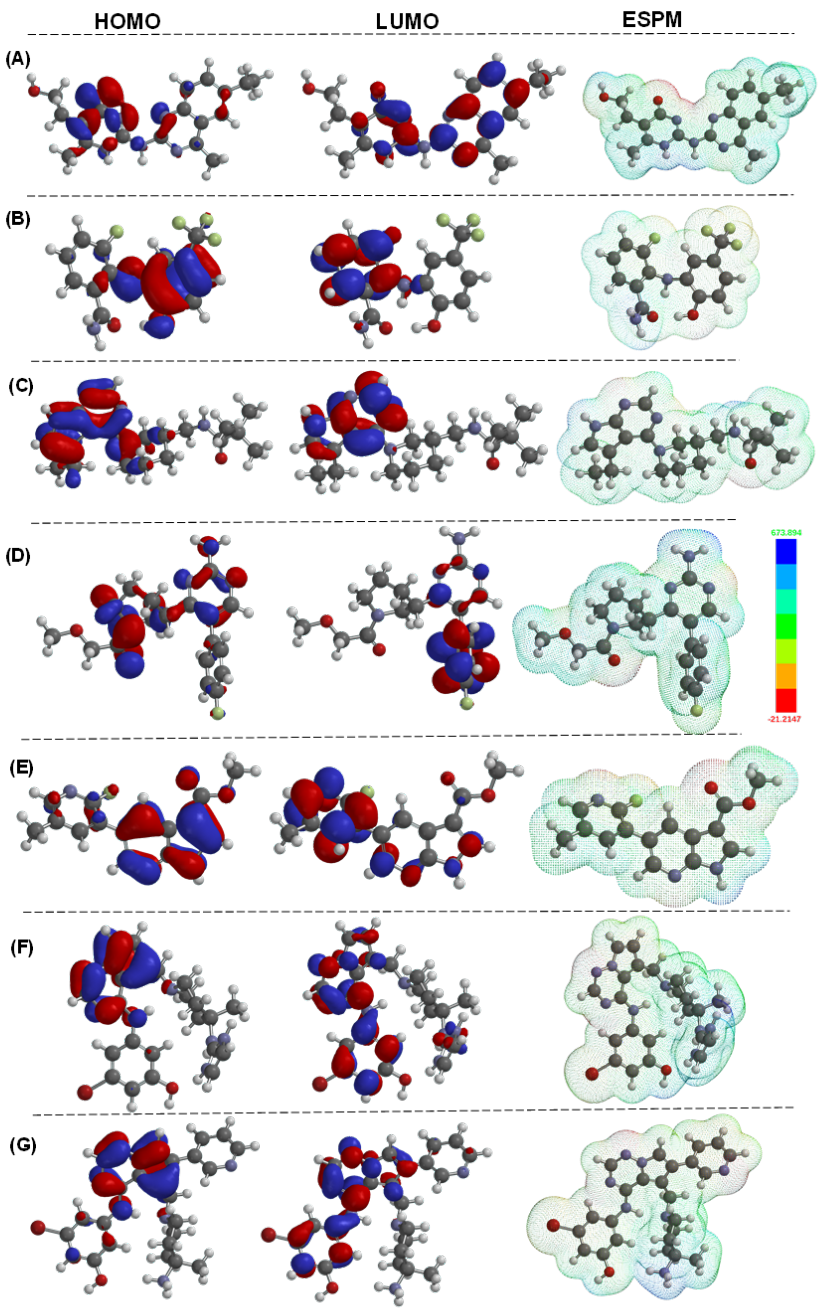

Figure S3. HOMO, LUMO orbitals and electrostatic potential map of shortlisted compounds ZINC000009936617, ZINC001209984530, ZINC000892508112, ZINC000892508112, ZINC00012184325, ZINC001243164470, LD10, and LD51 (A-G, respectively). The Calculations were performed by the Spartan'20 package with B3LYP/6-31G* in the gas phase. The graphs were produced with assistance from Spartan20 software (V1.0.0) package [2].

Table S1. The selected hypothesis and the validation of E-pharmacophore model.

PDB: ID	Hypothesis	EF1%	BEDROC (alpha=160.9)	BEDROC (alpha=20.0)	BEDROC (alpha=8.0)	ROC	AUAC	Ave Outranking Decoys	Total Actives	Ranked Actives	RIE
**4BTK**	AADHRRR	4.1	0.403	0.362	0.547	0.89	0.86	32.7	27	27	3.58
**4BTM**	AADRRR	8.2	0.672	0.675	0.719	0.76	0.81	7.86	27	21	6.67
**4NFN**	ADDHRR	11.6	0.91	0.929	0.919	0.94	0.87	4.7	27	23	9.24
**7JXX**	AADDDHRRR	12.3	1.00	0.952	0.927	0.92	0.89	1.8	27	25	9.40
**7JXY**	AAADDDHRRR	12.3	0.99	0.892	0.923	0.98	0.94	4.81	27	27	8.81

Table S2. The measurement of distances between e-pharmacophore hypothesis features in Å.

Pharmacophore model	Distance from A to A (Å)	Distance from A to D (Å)	Distance from A to H (Å)	Distance from A to R (Å)	Distance from D to D (Å)	Distance from D to H (Å)	Distance from D to R (Å)	Distance from H to R (Å)	Distance from R to R (Å)
**4BTK-DTQ: AADHRRR**	A1-A2: 2.38	A1-D6: 7.12<br>A2-D6: 8.89	A1-H8: 6.92<br>A2-H8: 4.98	A1-R10: 1.38<br>A1-R11: 3.68<br>A1-R12: 4.98<br>A2-R10: 1.38<br>A2-R11: 2.76<br>A2-R12: 6.38		D6-H8: 11.02	D6-R10: 8.89<br>D6-R11: 7.66<br>D6-R12: 3.26	H8-R10: 5.55<br>H8-R11:3.67<br>H8-R12:8.30	R10-R11: 2.41<br>R10-R12: 5.08<br>R11-R12: 5.01
**4BTM-F8E: AADRRR**	A1-A4: 8.66	A1-D5: 2.81<br>A4-D5: 11.00		A1-R8: 2.57<br>A1-R9: 1.44<br>A1-R10: 6.42<br>A4-R8: 9.21<br>A4-R9: 7.65<br>A4-R10: 3.77			D5-R8: 2.17<br>D5-R9: 3.44<br>D5-R10: 8.14		R8-R9: 2.13<br>R8-R10: 6.16<br>R9-R10: 5.10
**4NFN-2KC: ADDHRR**		A1-D4: 9.23<br>A1-D5: 4.74	A1-H8: 8.76	A1-R10: 2.54<br>A1-R11: 1.43	D4-D5: 5.81	D4-H8: 6.58<br>D5-H8: 4.63	D4-R10: 9.09<br>D4-R11: 7.95<br>D5-R10: 3.52<br>D5-R11: 3.34	H8-R10: 7.07<br>H8-R11: 7.55	R10-R11: 2.11
**7JXX-VP7: AADDDHRRR**	A1-A3: 10.16	A1-D4: 10.51<br>A1-D5: 3.25<br>A1-D6: 2.53<br>A3-D4: 0.91<br>A3-D5: 7.32<br>A3-D6: 8.96	A1-H8: 4.02<br>A3-H8: 10.11	A1-R9: 5.50<br>A1-R10: 1.40<br>A1-R11: 6.42<br>A3-R9: 8.25<br>A3-R10: 9.50<br>A3-R11: 6.13	D4-D5: 7.74<br>D4-D6: 9.43<br>D5-D6: 1.76	D4-H8: 10.18<br>D5-H8: 5.57<br>D6-H8: 6.02	D4-R9: 8.19<br>D4-R10: 9.77<br>D4-R11: 6.03<br>D5-R9: 5.66<br>D5-R10: 3.31<br>D5-R11: 5.42<br>D6-R9: 6.74<br>D6-R10: 3.33<br>D6-R11: 6.90	H8-R9: 2.44<br>H8-R10: 2.74<br>H8-R11: 4.44	R9-R10: 4.10<br>R9-R11: 2.18<br>R10-R11: 5.19

**7JXY-VSY: AAADDDHRRR**	A1-A2: 7.75<br>A1-A5: 5.33<br>A2-A5: 10.23	A1-D6: 5.20<br>A1-D7: 6.52<br>A1-D8: 8.10<br>A2-D6: 10.69<br>A2-D7: 3.26<br>A2-D8: 2.56<br>A5-D6: 0.97<br>A5-D7: 7.44<br>A5-D8: 9.15	A1-H9: 5.79<br>A2-H9: 8.19<br>A5-H9: 3.12	A1-R10: 3.45<br>A1-R11: 6.49<br>A1-R12: 1.39<br>A2-R10: 5.34<br>A2-R11: 1.40<br>A2-R12: 7.75<br>A5-R10: 8.15<br>A5-R11: 9.47<br>A5-R12: 6.10	D6-D7: 7.99<br>D6-D8: 7.44<br>D7-D8: 1.76	D6-H9: 4.03<br>D7-H9: 5.34<br>D8-H9: 6.88	D6-R10: 8.26<br>D6-R11: 9.86<br>D6-R12: 6.13<br>D7-R10: 5.65<br>D7-R11: 3.30<br>D7-R12: 5.56<br>D8-R10: 6.68<br>D8-R11: 3.34<br>D8-R12: 7.01	H9-R10: 7.41<br>H9-R11: 7.66<br>H9-R12: 5.88	R10-R11: 3.94<br>R10-R12: 2.18<br>R11-R12: 5.14

Table S3. Distance between the features of cavity pharmacophore hypothesis.

Pharmacophore Model	Distance from A to D (Ǻ)	Distance from A to A (Ǻ)	Distance from A to R (Ǻ)	Distance from D to R (Ǻ)	Distance from D to D (Ǻ)	Distance from R to R (Ǻ)
AADDDRR	4.06	6.89	3.45	8.71	5.93	4.79
	6.65		7.10	4.00	10.15	
	8.66		4.91	3.66	5.86	
	8.78		2.45	5.49		
	4.93			7.34		
	4.46			6.35		

Table S4. The top three compounds were retrieved after the pharmacophore model validation and docking.

No.	Fitness Score	XP GScore	ZINC ID
1.	1.545	-8.146	ZINC000892508112
2.	1.542	-8.014	ZINC000012184325
3.	1.506	-7.415	ZINC001243164470

Table S5. Validation of cavity pharmacophore hypothesis.

Pharmacophore Hypothesis	BEDROC(α=8.0)	BEDROC (α=20.0)	BEDRC (α=160.0)	ROC	RIE	AUAC
AADDDRR	0.980	0.980	1.000	0.80	4.63	0.82

Table S6. Lipinski RO5 of drug likeliness and ADME/T properties of the shortlisted compounds from ZINC database and Co-Crystal ligand interpreting pharmacological parameter.

Compound S. No.	CNS	MW	Donor HB	Accept HB	Rotatable bond	SASA	FOSA	FISA	PISA	WPSA	%HOA
Co-crystal-2KC	0	528.578	7	11.7	6	775.616	554.187	154.202	0	67.228	6.034
ZINC000095101333	-2	344.41	2	5.75	3	593.336	334.256	166.643	92.437	0	85.692
ZINC000009936617	-2	339.396	3	7.7	6	656.553	356.24	165.458	134.856	0	81.784
ZINC001209984530	-1	314.239	3	2.75	4	556.957	0.665	137.075	245.306	173.911	95.049
ZINC000892508112	0	343.471	2	5	4	628.767	439.205	87.489	102.073	0	100
ZINC000012184325	1	313.358	1	6.5	5	632.157	307.82	90.059	234.278	0	86.668
ZINC001243164470	-1	285.277	0	3	2	552.881	187.193	137.301	190.02	38.366	94.216

ADMET

	QPpolrz	QPlog PC16	QPlogPoct	QPlog Pw	QPlog Po/w	QPlogS	CIQPlogS	QPlogHERG	QPPCaco	QPlog BB	QPPMDCK	QPlog Kp
Co-crystal-2KC	6.034	6.034	6.034	6.034	6.034	6.034	6.034	6.034	6.034	6.034	6.034	-7.911
ZINC000095101333	85.692	85.692	85.692	85.692	85.692	85.692	85.692	85.692	85.692	85.692	85.692	-3.977
ZINC000009936617	81.784	81.784	81.784	81.784	81.784	81.784	81.784	81.784	81.784	81.784	81.784	-3.518
ZINC001209984530	95.049	95.049	95.049	95.049	95.049	95.049	95.049	95.049	95.049	95.049	95.049	-2.798
ZINC000892508112	100	100	100	100	100	100	100	100	100	100	100	-2.388
ZINC000012184325	86.668	86.668	86.668	86.668	86.668	86.668	86.668	86.668	86.668	86.668	86.668	-3.937
ZINC001243164470	94.216	94.216	94.216	94.216	94.216	94.216	94.216	94.216	94.216	94.216	94.216	-3.189

Table S7. Lipinski RO5 of drug likeliness and ADME/T properties of the ligands were compared to reference molecules, interpreting pharmacological parameters and predicted using the QikProp module of Schrodinger.

Compounds No.	R1	XP GScore	CNS	MW	Donor HB	AccptHB	Rotatable bond	SASA	FOSA	FISA	PISA	WPSA
LD7	from imidazole 1	-10.368	-1	497.396	4	7.75	7	721.759	170.773	140.954	332.717	77.314
LD10	from imidazolium 3	-9.978	0	497.396	4	7.75	7	712.68	167.572	137.571	330.225	77.311
LD51	from pyridine 2	-9.873	0	508.42	4	8.25	6	749.145	182.705	167.342	321.774	77.325
LD55	from imidazole 3	-9.793	-1	497.396	5	8.25	6	722.466	184.845	192.179	268.146	77.296
LD75	from benzenesulfonamide 2	-10.101	-2	571.491	4	10.75	8	721.403	183.74	194.831	344.128	77.703

ADMET

	R1	QPpolrz	QPlog PC16	QPlogPoct	QPlog Pw	QPlog Po/w	QPlogS	CIQPlogS	QPlogHERG	QPPCaco	QPlog BB	QPPMDCK	QPlog Kp	%HOA
LD7	from imidazole 1	45.557	15.343	26.126	16.653	3.1	-4.355	-6.168	-6.946	113.797	-0.708	138.531	-4.336	84.864
LD10	from imidazolium 3	45.363	15.242	25.83	16.591	3.105	-4.21	-6.168	-6.796	122.522	-0.645	150.04	-4.282	87.428
LD51	from pyridine 2	48.352	15.995	27.244	17.537	2.527	-3.61	-5.214	-7.743	25.951	-0.551	18.329	-7.019	53.017
LD55	from imidazole 3	45.669	15.495	27.689	18.742	1.794	-3.054	-4.827	-7.363	29.274	-0.772	10.195	-7.666	60.278
LD75	from benzenesulfonamide 2	51.979	17.462	29.902	20.021	2.127	-3.432	-5.52	-8.002	28.752	-1.005	9.626	-7.255	66.273

The minimal ranges for various molecular properties are as follows: Molecular Weight (MW) is between 130.0 to 725.0; Acceptor Hydrogen Bonds (accPthB) range from 2.0 to 20.0; the number of Rotatable Bonds (rotor) is from 0.0 to 15.0; log P for octanol/water (logP o/w) spans -2.0 to 6.5; log S for aqueous solubility (logS) ranges from -6.5 to 0.5; log S conformation independent (CIlogS) also spans -6.5 to 0.5; log BB for brain/blood (logBB) is between -3.0 and 1.2; Hydrophilic solvent accessible surface area (FISA) ranges from 7.0 to 700.0; log Kp for skin permeability (log Kp) is provided in cm/hr; Carbon Pi solvent accessible surface area (PISA) ranges from 0.0 to 450.0; log Khsa for serum protein binding (log Khsa) spans -1.5 to 1.5; the maximum number of Lipinski Rule of 5 Violations (RO5) is 4; vdW Polar surface area (PSA) ranges from 7.0 to 200.0; Human Oral Absorption in the GI tract is noted as poor if under 25%; Molecular Volume (volume) spans from 500.0 to 2000.0 Å^3; Apparent Caco-2 Permeability is considered poor below 25 nm/sec and excellent above 500 nm/sec; Donor Hydrogen Bonds (donorHB) range from 0.0 to 6.0; Apparent MDCK Permeability is poor below 25 nm/sec and excellent above 500 nm/sec; CNS permeability (CNS) ranges from -5.00 to 3.00.

1 Schrödinger. Maestro. *Schrödinger, LLC, New York, NY* **2023** (2023).

2 Hehre, W. J. & Huang, W. W. *Chemistry with computation: an introduction to SPARTAN*. (Wavefunction, Incorporated, 1995).
